# Increased Indoleamine 2,3-Dioxygenase and Quinolinic Acid Expression in Microglia and Müller Cells of Diabetic Human and Rodent Retina

**DOI:** 10.1167/iovs.17-21654

**Published:** 2017-10

**Authors:** Ping Hu, Nicholas H. Hunt, Frank Arfuso, Lynn C. Shaw, Mohammad Nasir Uddin, Meidong Zhu, Raj Devasahayam, Samuel J. Adamson, Vicky L. Benson, Tailoi Chan-Ling, Maria B. Grant

**Affiliations:** 1 Department of Anatomy, Bosch Institute, University of Sydney, New South Wales, Australia; 2 Department of Ophthalmology, the Eugene and Marilyn Glick Eye Institute, Indiana University, Indianapolis, Indiana, United States; 3 Department of Pathology, Bosch Institute, University of Sydney, New South Wales, Australia; 4 Stem Cell & Cancer Biology Laboratory, School of Biomedical Sciences, Curtin Health Innovation Research Institute, Curtin University, Perth, Australia; 5 Lions New South Wales Eye Bank, New South Wales Organ and Tissue Donation Service, South Eastern Sydney Local Health District, New South Wales, Australia; 6 Save Sight Institute, Discipline of Clinical Ophthalmology and Eye Health, University of Sydney, New South Wales, Australia; 7 Department of Physiology, Faculty of Health and Medical Sciences, University of Auckland, Auckland, New Zealand; 8 Univeristy of Alabama, Birmingham, Alabama, United States

**Keywords:** indoleamine 2, 3-dioxygenase, quinolinic acid, microglia, neuronal loss

## Abstract

**Purpose:**

We investigated the relationship between inflammation, neuronal loss, and expression of indoleamine 2, 3-dioxygenase (IDO) and quinolinic acid (QUIN) in the retina of subjects with type 1 diabetes (T1D) and type 2 diabetes (T2D) and in the retina of rats with T1D.

**Methods:**

Retinas from T1D (*n* = 7), T2D (*n* = 13), and 20 age-matched nondiabetic human donors and from T1D (*n* = 3) and control rats (*n* = 3) were examined using immunohistochemistry for IDO, QUIN, cluster of differentiation 39 (CD39), ionized calcium-binding adaptor molecule (Iba-1, for macrophages and microglia), Vimentin (VIM; for Müller cells), neuronal nuclei (NeuN; for neurons), and UEA1 lectin (for blood vessels).

**Results:**

Based on morphologic criteria, CD39^+^/ionized calcium binding adaptor molecule 1(Iba-1^+^) resident microglia and CD39^−^/Iba-1^+^ bone marrow–derived macrophages were present at higher density in T1D (13% increase) and T2D (26% increase) human retinas when compared with controls. The density and brightness of IDO^+^ microglia were increased in both T1D and T2D human retinas. The intensity of QUIN^+^ expression on CD39^+^ microglia and VIM^+^ Müller cells was greatly increased in both human T1D and T2D retinas. T1D retinas showed a 63% loss of NeuN^+^ neurons and T2D retinas lost approximately 43% when compared with nondiabetic human retinas. Few QUIN^+^ microglia-like cells were seen in nondiabetic retinas, but the numbers increased 18-fold in T1D and 7-fold in T2D in the central retina. In T1D rat retinas, the density of IDO^+^ microglia increased 2.8-fold and brightness increased 2.1-fold when compared with controls.

**Conclusions:**

Our findings suggest that IDO and QUIN expression in the retinas of diabetic rats and humans could contribute to the neuronal degeneration that is characteristic of diabetic retinopathy.

Indoleamine 2,3-dioxygenase (IDO) is the first and rate-limiting enzyme of tryptophan catabolism via the kynurenine pathway (KP).^1^ The KP represents the major catabolic route of tryptophan (TRP), an essential amino acid that has various important biological functions. TRP is a source of nicotinamide adenine nucleotide (NAD^+^), a cofactor in cellular respiration and energy production that plays an important role in DNA repair and transcriptional regulation.^2^ IDO has immunologic functions. In the initial stage of some infectious diseases it can, by depleting TRP, inhibit the growth of the pathogens^3^ and thereby disease progression. In the later stages, IDO may be involved in regulating immune responses and creating immune tolerance, acting as a protective feedback mechanism against an overzealous T-cell response.^4^ IDO consists of IDO-1 and IDO-2, proteins that have similar enzymatic actions in the human and mouse.^5,6^ IDO expression is low in the normal central nervous system (CNS), but increases greatly in inflammatory conditions such as cerebral malaria.^7^ The increase in IDO expression is due to proinflammatory cytokines, especially interferon gamma (IFN-γ).^8,9^ With increased IDO expression, tissue levels of KP downstream products such as quinolinic acid (QUIN) typically rise.

QUIN is an agonist of the *N*-methyl-d-aspartate receptor. Under normal physiological conditions, QUIN may modulate some local CNS events. However, under pathologic conditions, increased QUIN can lead to neuronal dysfunction and death via several processes. The main mechanism in the CNS is via overactivation of *N*-methyl-d-aspartate receptors, leading to increased intracellular calcium concentration and glutamate release, followed by mitochondrial dysfunction and adenosine triphosphate (ATP) exhaustion (energy depletion), and free radical formation and oxidative damage (reviewed in Ref. 10). Astrocyte dysfunction and gliotoxicity, blood–brain damage, and inflammation induced by QUIN also are implicated in its neurotoxicity.^11^ Thus, activation of the KP in the CNS may damp down inflammatory processes but also damage neurons via the production of QUIN.^12^

Diabetic retinopathy (DR) is a complex disorder that involves both systemic and retinal tissue-specific initiating factors and cell types. A number of hyperglycemia- and dyslipidemia-activated pathways leading to retinal endothelial cell and neural cell dysfunction have been identified.^3–21^ Thus, the loss of neurons is also observed as part of the pathophysiology^22–29^; ultimately the summation of these aberrant events can lead to vision loss and even blindness.

Activation of the KP has been implicated in both the causation^30–34^ and complications^35–38^ of diabetes. The KP downstream products, kynurenine and 3-hydroxykynurenine, were found to be increased in the serum of DR individuals.^39^ In streptozotocin-induced diabetes in rats, the levels of IDO activity and mRNA in the lenses were raised when compared with those of nondiabetic animals, and oxidative stress markers, for example, thiobarbituric acid-reacting substances, were also increased when compared with control lenses.^35^

We therefore hypothesized that neuronal loss in DR is mediated via products of inflammation, including metabolites of TRP derived from the KP. In this study, the expression of markers of inflammation and retinal damage were evaluated alongside the expression of IDO and QUIN in a series of retinas from normal, T1D, and T2D human subjects and in a rat model of T1D.

## Materials and Methods

### Human Eyes

We used 40 human adult eyes (20 nondiabetic, 7 T1D; 13 T2D; one eye represents one subject), aged 46 to 78 years, obtained from the Lions NSW Eye Bank in accordance with the Declaration of Helsinki for the Use of Human Tissue. This study was approved by the Human Research Ethics Committee of the University of Sydney (Approval 15190). Cause of death for the donors was predominantly cardiovascular diseases or cancer. Among the eyes, 7 were from donors with a history of T1D, and 13 with a history of T2D. All eyes were enucleated within 12 to 24 hours following death. After removing the corneas and the anterior segments, the eyes were fixed in 2% w/v paraformaldehyde overnight and then transferred into PBS. The eyes were examined using immunohistochemistry.

### Animals and Experimental Diabetes

Male Sprague-Dawley rats were obtained from Animal Resource Center in Perth and housed in the institutional animal care facilities at the University of Sydney. The Animal Ethics Committee of the University of Sydney approved all animal protocols, and complied with the ARVO Statement for the Use of Animals in Ophthalmic and Vision Research.

Experimental diabetes was induced as previously described.^40^ Briefly, Sprague-Dawley rats aged 7 to 10 weeks were rendered diabetic with a single intraperitoneal injection of streptozotocin (STZ, 90 mg/kg) freshly dissolved in citrate buffer (pH 4.5). Development of diabetes (defined by blood glucose greater than 15 mmol/L) was verified 1 week after the first STZ injection (Accu-Chek Peforma blood glucometer; Roche Diagnostics GmbH, Mannheim, Germany). Diabetes was confirmed by blood glucose levels greater than 15 mmol/L on repeated testing.^41^ To sustain the rats for a longer time, insulin was administered to diabetic rats every 3 days based on body weight and blood glucose levels (the mortality rate in the STZ model at 8 weeks was 8.3%). Three animals were examined in each cohort, control and diabetic.

### Immunofluorescence Histochemistry on Retinal Whole Mounts and Transverse Sections

The entire adult human retinal flat mount is too large for microscopic examination, and as different combinations of antibodies were required for the same retina, the whole retina was cut into several pieces. Approximately 80% of the pieces were from the nasal side (between 12 to 3 o'clock), and 20% were from the temporal side (between 10 to 12 o'clock) to avoid the fovea region.^42^ Each piece contained the central, middle, and peripheral regions. All images and data were collected from the middle-peripheral region. The pieces of flat mounted retina or sections on slides were stained with antibodies to indoleamine 2,3-dioxygenase 1 (IDO1; LS-B1746, LSBio, Seattle, WA, USA), quinolinic acid (QUIN, Ref. 21203002; apDia, Turnhout, Belgium), kynurenine 3 monooxygenase (catalog number 10698-1-AP; proteintech, Rosemont, IL, USA), Iba-1 (Wako, Osaka, Japan) for visualization of microglia/macrophages,^43^ CD39 (22A9; Novocastra/Leica Microsystems, Bannockburn, IL, USA) for visualization of resident microglia and blood vessels, Ulex europaeus lectin (UEA lectin, L9006; Sigma-Aldrich Corp., St. Louis, MO, USA) for blood vessels, biotinylated *Griffonia simplicifolia* (*Bandeiraea*) isolectin B4 (GS Lectin, L3795; Sigma-Aldrich Corp.) for rat microglia/macrophages and blood vessels, monoclonal anti-vimentin−Cy3 antibody produced in mouse (C-9080, used at 1:200; Sigma-Aldrich Corp.) was used to identify Müller cells, and neuronal nuclei (NeuN, MAB 377; Chemicon, Temecula, CA, USA) for neurons. The tissues were washed and transferred to secondary antibodies conjugated with either Alexa594 or Alexa488 (Invitrogen-Molecular Probes, Carlsbad, CA, USA).

### Confocal Microscopy

Imaging was carried out using a ZEISS LSM 510 Meta confocal microscope at the Bosch Advanced Microscopy Facility (University of Sydney). Images were captured with the ZEISS LSM 510 acquisition software (Carl Zeiss, North Ryde, NSW, Australia). Z-stack images were collected. The optimal interval, pinhole size, and optical depth parameters were consistently maintained as required for the 20 × 0.8NA and 40 × 0.75NA, using an image frame size of 1024 × 1024 pixels. The laser lines were 405, 488, 561, and 633 nm. Scan speed and averaging remained consistent for all images captured in each experiment for both qualitative and quantitative purposes. Image analysis was performed using LSM 510 Meta 4.2 (Carl Zeiss) offline software and Adobe Photoshop CS6 version 12.0 software (Adobe Systems, San Jose, CA, USA) on an Apple (Cupertino, CA, USA) Macintosh computer .

### Quantitative Analysis of Iba-1^+^ Microglia/Macrophages, Iba-1^−^/CD39^+^ Resident Microglia, IDO^+^ or QUIN^+^ Microglia-Like Cell Densities and Intensities (Brightness), and IDO^+^ Blood Vessel and QUIN^+^ Müller-Like Cell Intensities

Confocal images (20×) of the flat mounted retinas were analyzed to obtain the data of Iba-1^+^ microglia/macrophages and Iba-1^-^/CD39^+^ resident microglia, IDO^+^ or QUIN^+^ Microglia-like cell densities and intensities as well IDO^+^ blood vessel and QUIN^+^ Müller-like cell intensities. In each sample, three or more images were taken from different regions of the retinas. The positive cells were determined by their specific antibody labeling and unique morphology, and the images were analyzed using ImageJ software (NIH Research Services Branch, http://rsb.info.nih.gov/ij/index.html).

### Quantitative Analysis of NeuN^+^ Neuron Densities

Confocal images (40×, 10 mm from the optic nerve head) of the retinal sections were analyzed to obtain the densities of NeuN^+^ neurons in the ganglion cell layer from diabetic and control human retinas. For each individual, three or more images were taken from different sections for analysis. The positive cells were determined by their specific antibody labeling and unique morphology. The density of positive cells was quantified manually using ImageJ software.

### Statistical Analysis

The density and fluorescence intensity of positive cells was normalized by dividing by the mean values of age-matched controls in the same region or tissue and expressed as the percentage of relative densities or fluorescence intensity for each retina.^40^ All data are shown as mean values ± SEM. Statistical differences between two groups, for example, control compared to T1D, or T1D compared to T2D, were determined by applying ANOVA (for comparison among three groups) and 1-tailed Student's *t*-tests (for comparison between groups) for unpaired groups with equal variance. A *P* value of less than 0.05 (*P* < 0.05) was considered statistically significant.

## Results

### Increased Iba-1^+^ Microglia/Macrophage Density and Decreased CD39 Expression in T1D and T2D Human Retinas

**Figure 1:**
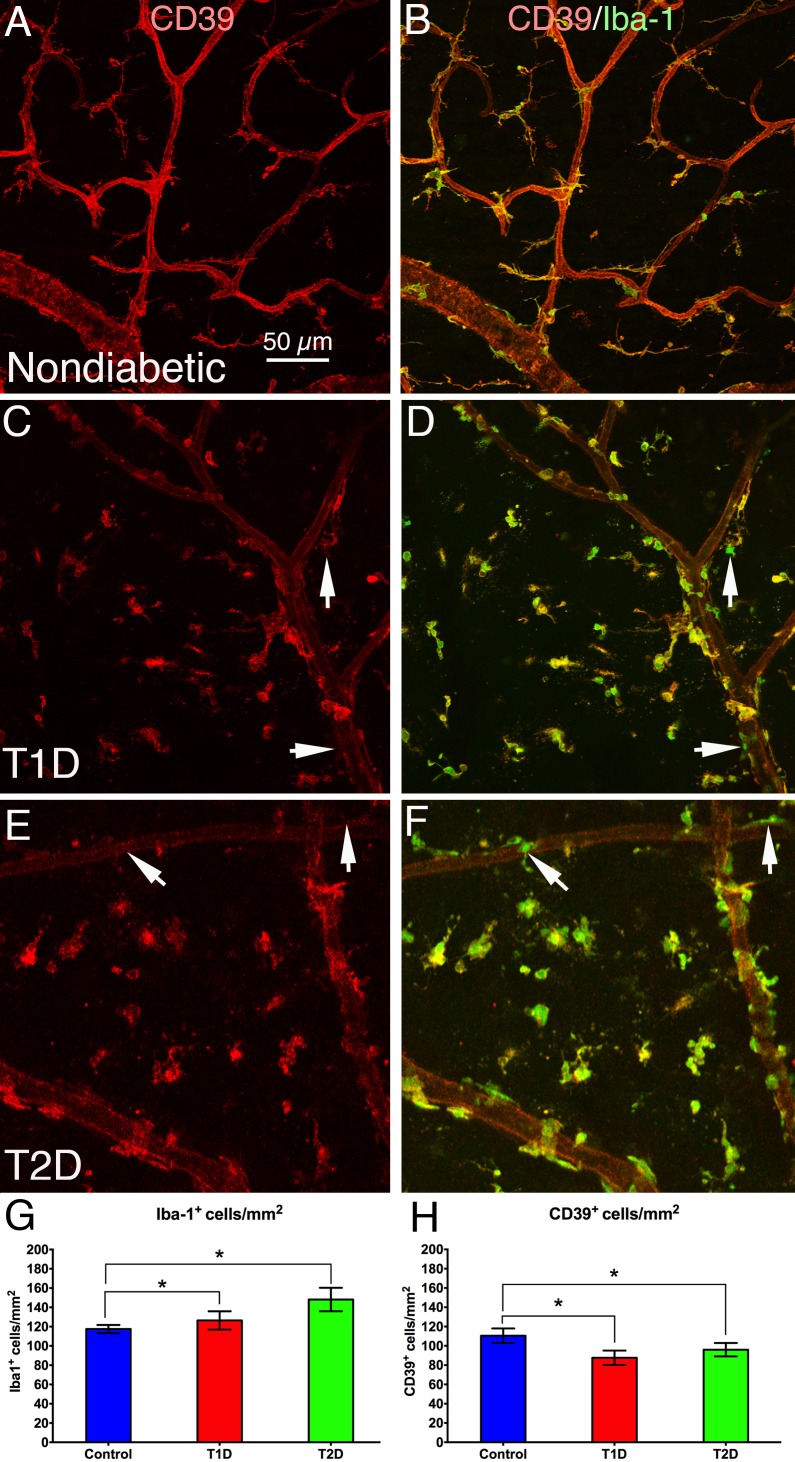
Retinal flat mounts from nondiabetic (A, B), T1D (C, D), and T2D (E, F) human retinas, double stained with CD39 (red) and Iba-1 (green). A, C, and E show only CD39 labeling, whereas B, D, and F show both Iba-1 and CD39 staining. The images show that the density of Iba-1^+^ microglia was increased, whereas bright CD39^+^ microglia and CD39 expression on blood vessels decreased in human T1D and T2D retinas, which suggests that inflammation is occurring in human T1D and T2D retinas. Arrows indicate the presence of some CD39^−^/Iba-1^+^ bone marrow–derived macrophages (CD39 is only expressed on resident microglia, suggesting a bone marrow origin of these cells). (G) Quantitative analysis shows Iba-1^+^ microglia density was significantly higher in both T1D (13%) and T2D (26%; *P < 0.05) retinas when compared with nondiabetic controls. N = 5 to 10 samples/group, and there was no significant difference between T1D and T2D. (H) Quantitative analysis shows that the bright CD39^+^ microglia density was significantly lower in both T1D (21%) and T2D (13%; *P < 0.05) retinas when compared with nondiabetic controls. N = 5 to 15 samples/group, and there was no significant difference between T1D and T2D. Calibration in A: for A–F. Arrows in E, F indicate CD39^−^/ Iba-1^+^ bone marrow–derived macrophages.

**Figure 2:**
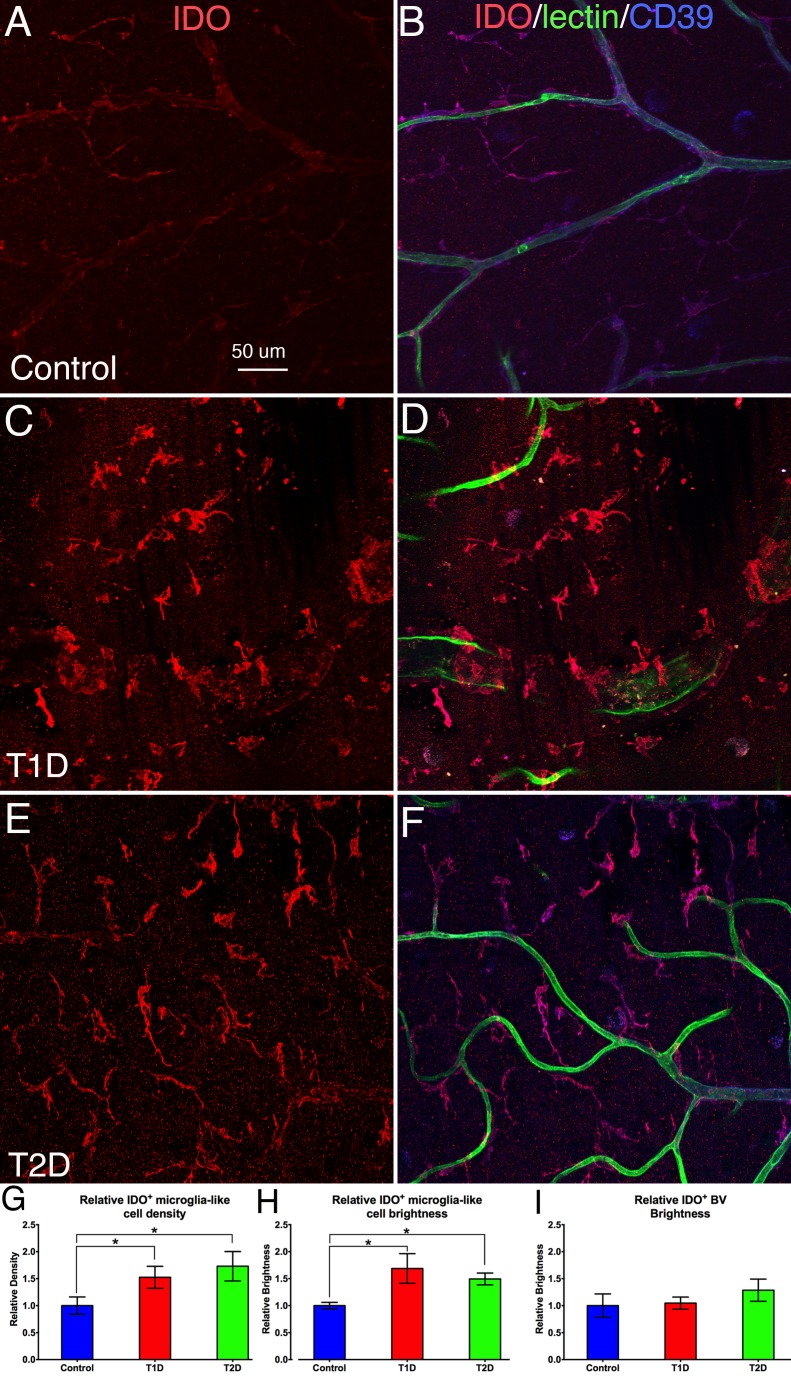
Retinal flat mounts from nondiabetic (A, B), T1D (C, D), and T2D (E, F) human retinas, triple stained with IDO (red), UEA lectin (green), and CD39 (blue). A and B, C and D, E and F are the same images, with A, C, and E showing only IDO labelling. The images in A and B show weak IDO^+^ expression on CD39^+^ microglia and CD39^+^/UEA lectin^+^ blood vessels. In T1D and T2D, IDO^+^ microglia increased in density and brightness, but IDO expression on blood vessels did not change. (G) Quantitative analysis shows IDO^+^ microglia-like cell density was significantly higher in both T1D (53%) and T2D (56%; *P < 0.05) retinas when compared with nondiabetic controls. N = 4 to 6 samples/group, and there was no difference between T1D and T2D. (H) Quantitative analysis shows IDO^+^ cell brightness (intensity) was significantly higher both in T1D (68%) and T2D (54%; *P < 0.05) retinas when compared with nondiabetic controls. N = 4 to 6 samples/group, and there was no difference between T1D and T2D. (I) Quantitative analysis shows that the bright IDO^+^ expression on blood vessel endothelial cells (BVECs) was similar among the T1D, T2D, and nondiabetic control groups. (N = 4–6 samples/group). Calibration in A: for A–F.

The fluorescence intensity and the density of Iba-1^+^ microglia/macrophages increased significantly (*P* < 0.05) in T1D and T2D retinas when compared with nondiabetic retinas (Fig. 1B compared to 1D, 1F). Quantitative analysis (Fig. 1G) showed that there was no significant difference of CD39^+^ and Iba-1^+^ cell densities between T1D and T2D. When compared with nondiabetic retinas (Fig. 2A), the bright CD39^+^ microglia density in the T1D (Fig. 1C) and T2D (Fig. 1E) retinas was decreased significantly (*P* < 0.05, see Fig. 1H), with no significant difference between T1D and T2D retinas. The CD39^+^/Iba-1^+^ microglia in the diabetic retinas often displayed shorter processes (Figs. 1D, 1B–F), indicating that these microglia were in an activated state. Some Iba-1^+^ microglia/macrophages without CD39 expression were also detected (arrows in Figs. 1C–F), suggesting that the cells were bone marrow–derived macrophages (CD39 is only expressed on resident microglia).^44^

### Increased Density and Brightness of IDO^+^ Microglia Are Observed in Both T1D and T2D Human and Rat Retinas

**Figure 3:**
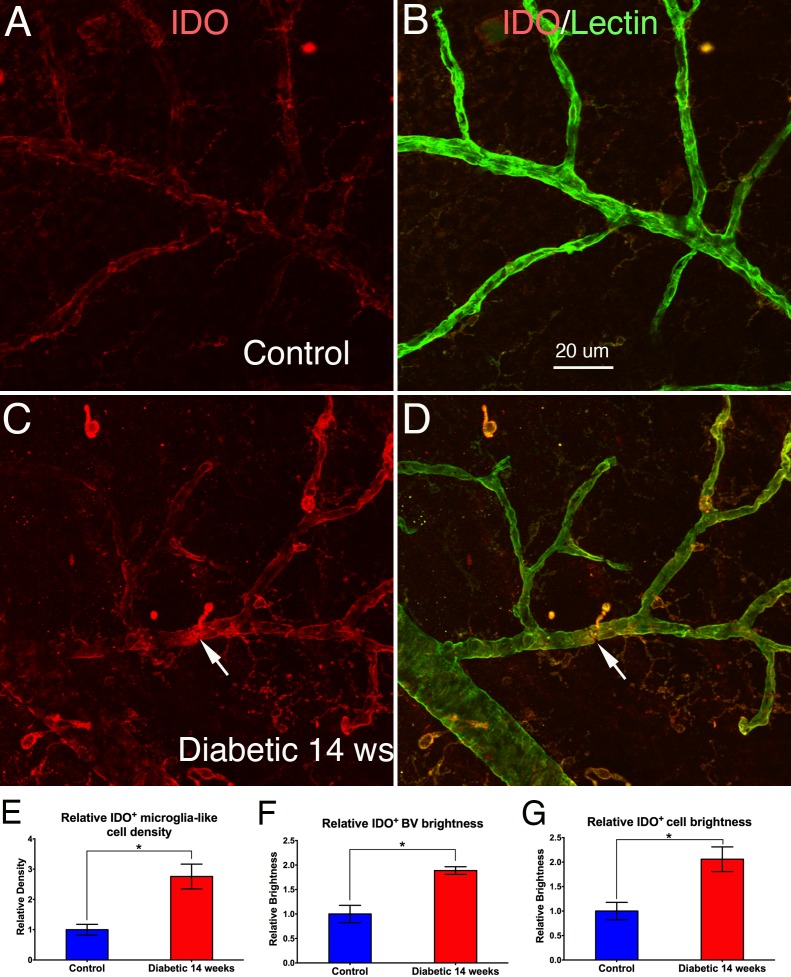
Retinal flat mounts from nondiabetic (A, B) and T1D (C, D) rat retinas, double stained with IDO (red) and GS lectin (green). A and B, C and D are the same images, with A and C only showing IDO labeling. The images in A and B show weak IDO^+^ expression on GS lectin^+^ microglia and GS lectin^+^ blood vessels. In T1D (C, D), IDO^+^/Lectin^+^ microglia (arrows) increased in density and brightness, and IDO^+^ labeling on blood vessels also increased when compared with control. (E) Quantitative analysis shows IDO^+^ microglia-like cell density was significantly higher in T1D (2.76 times; *P < 0.05) retinas when compared with nondiabetic controls. N = 3 samples/group. (F) Quantitative analysis shows IDO^+^ cell brightness (intensity) was significantly higher in T1D (2.06 times; *P < 0.05) retinas when compared with nondiabetic controls. (G) Quantitative analysis shows that the bright IDO^+^ expression on BVECs was significantly higher in T1D (1.88 times; *P < 0.05) retinas when compared with nondiabetic controls. Calibration in A: for A–D.

Microglia weakly positive for IDO were seen around CD39^+^/UEA lectin^+^ blood vessels in the nondiabetic retinas (Figs. 2A, 2B). All IDO^+^ microglia were CD39^+^, suggesting that they are resident retinal microglia. In the T1D (Figs. 2C, 2D) and T2D (Figs. 2E, 2F) retinas, IDO^+^ microglia increased in number and brightness (Fig. 2A compared to 2C, 2F). Quantitative analysis (Fig. 2G) showed the IDO^+^ microglia density to be significantly (*P* < 0.05) higher in T1D and T2D retinas when compared with nondiabetic retinas, with no significant difference between T1D and T2D. IDO immunostaining intensity on positive microglia (Fig. 3H) was significantly (*P* < 0.05) higher in T1D and T2D retinas when compared with nondiabetic retinas, with no difference between T1D and T2D. However, IDO immunostaining intensity on vascular endothelial cells (Fig. 2I) was similar in the three conditions.

In T1D rat retinas, IDO^+^ microglia increased in density and brightness, and IDO^+^ labeling on blood vessels also increased when compared with controls (Figs. 3A, 3B compared to 3C, 3D). Quantitative analysis showed IDO^+^ microglia-like cell density was significantly higher in T1D (2.76 times, *P* < 0.05) retinas when compared to nondiabetic retinas (Fig. 3E). IDO^+^ microglia-like cell brightness (intensity) was significantly greater in T1D (2.06 times, *P* < 0.05) retinas when compared with nondiabetic rats (Fig. 3F). The bright IDO^+^ expression on blood vessel endothelial cells was also significantly higher in T1D (1.88 times, *P* < 0.05) retinas when compared with nondiabetic retinas (Fig. 3G).

### QUIN^+^ Expression on Microglia-Like and Müller-Like Cells Is Greatly Increased in Human T1D and T2D Retinas

**Figure 4:**
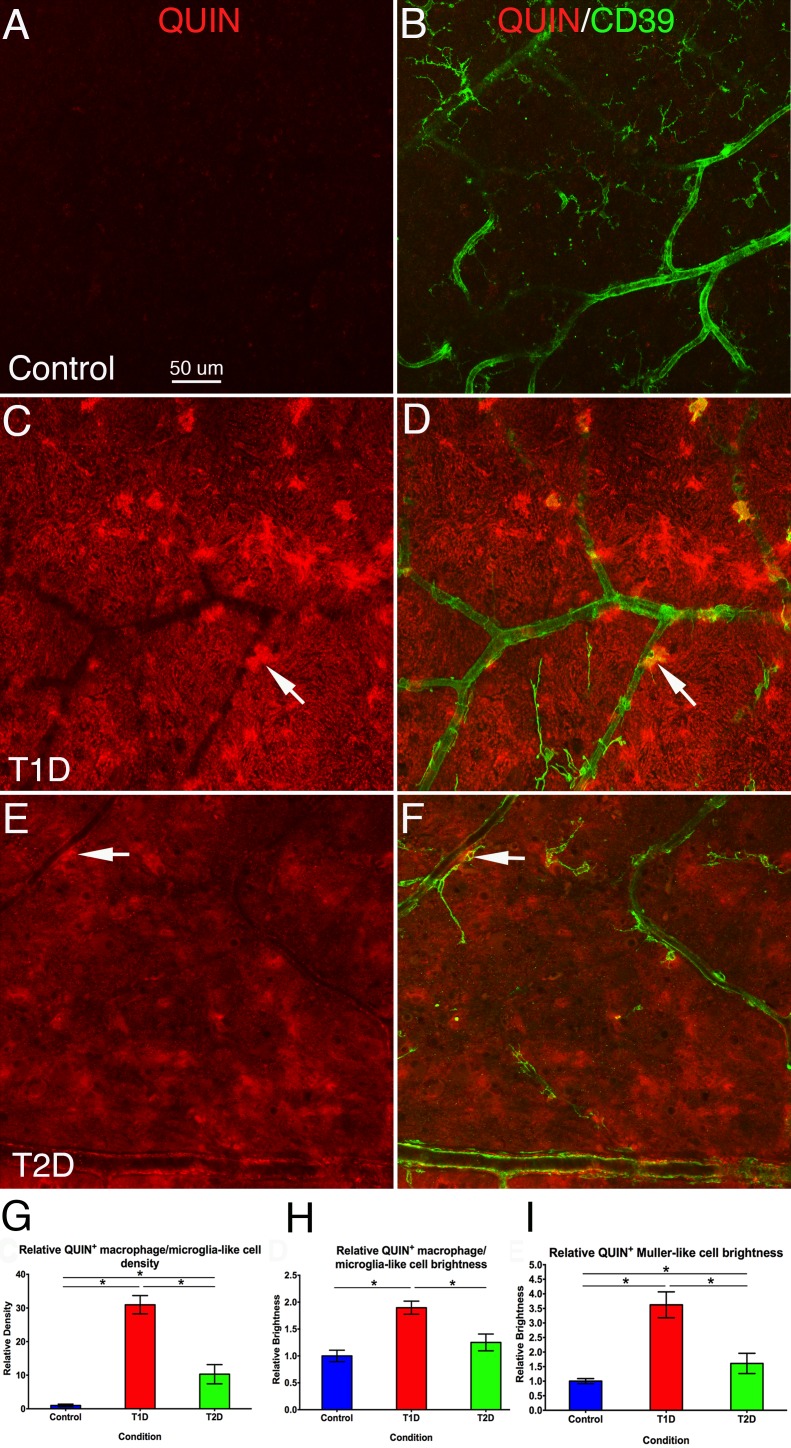
Retinal flat mounts from nondiabetic (A, B), T1D (C, D), and T2D (E, F) human retinas, double stained with QUIN (red) and CD39 (green). A and B, C and D, E and F show the same fields of view, but A, C, and E only show QUIN labeling. The images show a very weak level of QUIN^+^ microglia/macrophage in nondiabetic retinas, whereas the density of QUIN^+^ microglia/macrophages was increased greatly in T1D and to a lesser extent in T2D retinas. Some of the QUIN^+^ microglia/macrophages are CD39 positive (arrows). Besides QUIN^+^ microglia/macrophages, the retinal parenchyma also showed a high level of background QUIN^+^ expression. (G) Quantitative analysis shows QUIN^+^ microglia-like cell density was significantly higher in both T1D (31 times) and T2D (10.3 times; *P < 0.05) retinas when compared with nondiabetic controls (N = 4–6 samples/group), and significantly higher in T1D than T2D (*P < 0.05). (H) Quantitative analysis shows QUIN^+^ microglia-like cell brightness (intensity) was significantly higher in T1D (1.9 times) retinas when compared with nondiabetic controls and T2D. N = 4 to 6 samples/group (*P < 0.05), and there was no difference between nondiabetic controls and T2D. (I) Quantitative analysis shows QUIN^+^ Müller-like cell brightness (intensity) was significantly higher in T1D (3.6 times) and T2D (1.5 times; *P < 0.05) retinas when compared with nondiabetic controls, and significantly higher in T1D than T2D (*P < 0.05). N = 4 to 6 samples/group. Calibration in A: for A–F.

**Figure 5:**
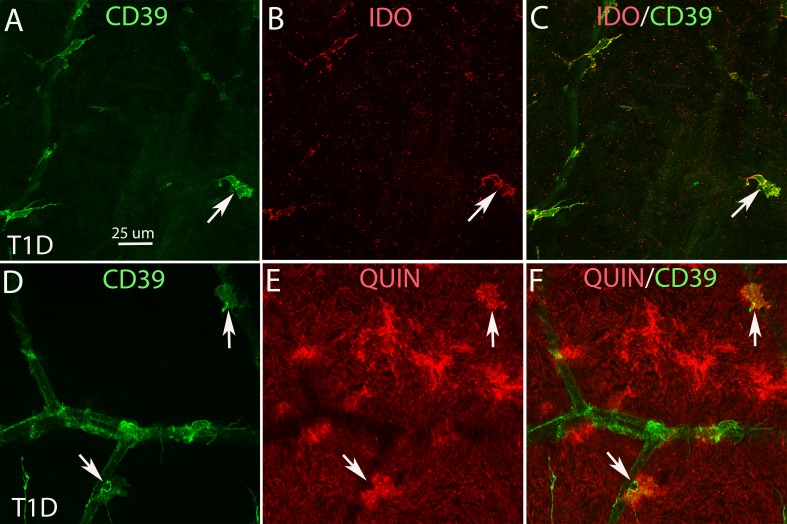
Retinal flat mounts from T1D (A–F) human retinas, double stained with CD39 (green) and IDO (red) or QUIN (red). A, B, and C and D, E, and F show the same fields of view. A and D only show CD39 labeling. B only shows IDO labeling. E shows only QUIN labeling. C is the merged image of A and B. F is the merged image of D and E. The images A–C show some CD39^+^ microglia (arrow in A) were IDO positive (arrows in B and C). The images D–F (higher magnification from Figs. 4C, 4D) show some CD39^+^ microglia are QUIN positive. Calibration in A: for A–F.

**Figure 6:**
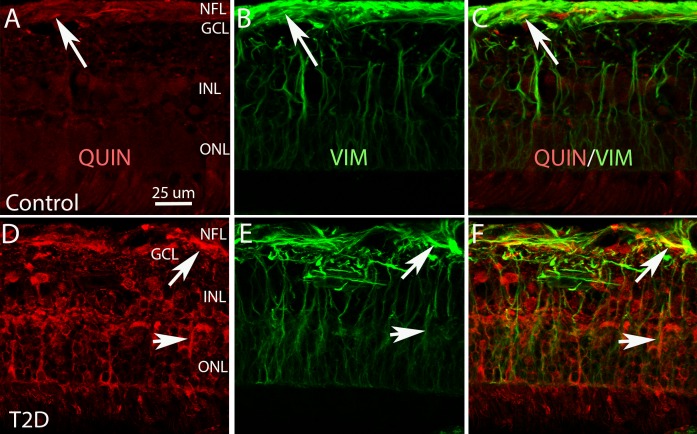
Middle-peripheral region of retinal cross sections from human nondiabetic (control, A–C) and T2D (D–F) retinas, double stained with QUIN (red) and VIM (green). A, B, and C and D, E, and F show the same fields of view. A and D only show QUIN labeling. B and E show only VIM labeling. C is the merged image of A and B. F is the merged image of D and E. The images A–C show a few QUIN^+^ fibers (arrow in A) are VIM positive (arrows in B and C). The images D–F show QUIN^+^ labeling increases in T2D retina, and many QUIN^+^ labeled (arrows in D) are VIM positive (arrows in E and F). Calibration in A: for A–F. GCL, ganglion cell layer; INL, inner nuclear layer; ONL, outer nuclear layer.

In 33% of nondiabetic human retinas (Figs. 4A, 4B), there were no QUIN^+^ cells. In the other 67% of nondiabetic retinas, there were very few weakly labeled QUIN^+^ cells (mean 0.64 ± 0.26/mm^2^). However, in T1D (Figs. 4C, 4D) and T2D (Figs. 4E, 4F), the QUIN^+^ cell number increased, especially in T1D (mean 30.96 ± 2.73/mm^2^; T2D, mean 10.3 ± 2, 86/mm^2^). Some of them were CD39^+^ microglia (arrows in Figs. 4C–F, Figs. 5D–F), and the CD39 negative QUIN^+^ cells also had microglial morphology. In addition to the increased number of QUIN^+^ microglia, QUIN^+^ labeling was evident on Müller-like cells in the parenchyma in human T1D and T2D retinas in flat mount preparations (Figs. 4A, 4B compared to 4C–F). The images from retinal sections confirmed that the QUIN^+^ labeling was on a few Müller cells in the nondiabetic retinas, but greatly increased in T2D retinas (Figs. 6A–C compared to 6D–F). Quantitative analysis confirmed that the density of QUIN^+^ microglia-like cells was significantly higher (*P* < 0.05) in both T1D and T2D retinas when compared with the nondiabetic retinas, and the density of QUIN^+^ microglia-like cells in T1D retinas was significantly (*P* < 0.05) greater than in T2D retinas (Fig. 4G). The brightness of QUIN^+^ microglia-like cells was significantly higher (*P* < 0.05) in T1D, but not T2D, retinas when compared with nondiabetic retinas (Fig. 4H). The brightness of QUIN^+^ Müller-like cells was significantly (*P* < 0.05) greater in both T1D and T2D retinas when compared with nondiabetic retinas, with it being significantly (*P* < 0.05) higher in T1D than in T2D (Fig. 4I). The localized QUIN expression on microglia-like and Müller cells is strongly suggestive of QUIN production by these cell types rather than it being of systemic origin.

### T1D Retinas Had Fewer NeuN^+^ Neurons When Compared With Nondiabetic Retinas

**Figure 7:**
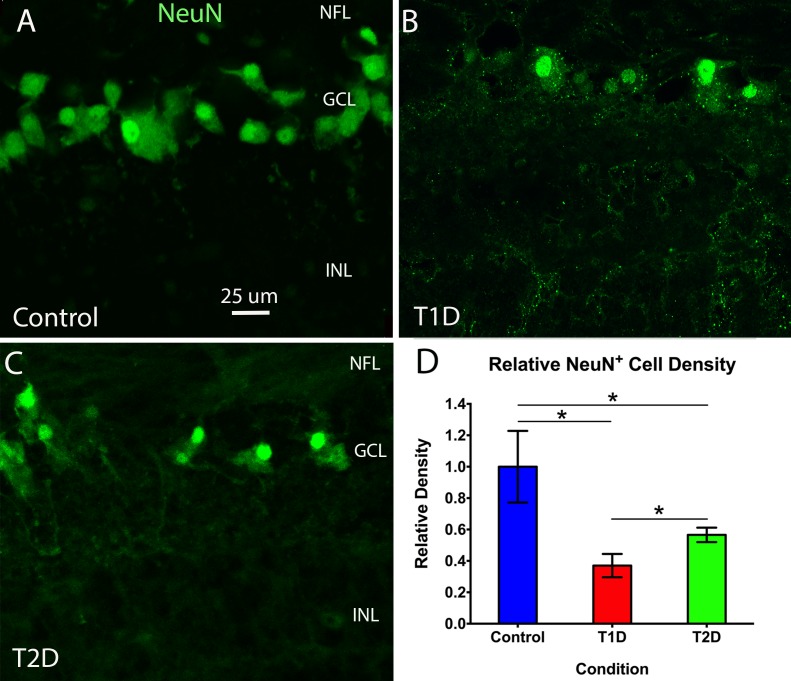
Middle-peripheral region of retinal cross-sections from nondiabetic (A), T1D (B), and T2D (C) retinas stained with NeuN in the ganglion cell layer. The images show many NeuN^+^ neurons in the ganglion cell layer of the nondiabetic retina (A). In the T1D (B) and T2D (C) retinas, there are far fewer NeuN^+^ neurons. (D) Quantitative analysis showed a marked decrease in the density of NeuN^+^ cells in T1D and T2D (*P < 0.05, 63% and 43% reduction, respectively) relative to controls (nondiabetic), and there was significantly less in T1D then in T2D (*P < 0.05). N = 3 to 4 samples/group. Calibration in A: for A–C.

Many NeuN^+^ neurons were seen in the ganglion layer in the nondiabetic retinas (Fig. 7A). In T1D (Fig. 7B) and T2D (Fig. 7C) retinas, there were fewer NeuN^+^ neurons in the ganglion cell layer. Quantitative analysis (Fig. 7D) showed that the NeuN^+^ cells were 63% fewer and 43% fewer relative to the nondiabetic retinas in T1D and T2D, respectively (*P* < 0.05), and there were significantly fewer in T1D than in T2D (*P* < 0.05).

## Discussion

Diabetes is strongly associated with a systemic proinflammatory state,^45,46^ including raised circulating levels of the proinflammatory cytokines interferon-gamma (IFN-γ) and TNF.^47–49^ These cytokines can activate IDO, and therefore the KP, in tissues, including the CNS.^50^ KP activity is known to downregulate immune and inflammatory processes, thereby acting as a protector of tissue structure and function.^51^ However, some CNS cells are susceptible to the cytotoxic effects of KP products such as QUIN, and this is believed to contribute to the manifestations of several neurologic disorders.^8,12,50,52^ Our data are consistent with such a process contributing to DR.

Microglia activation, increased IDO and QUIN expression, and loss of NeuN^+^ neurons were observed in the retinas from T1D and T2D human eyes when compared with age-matched controls. We propose that these changes are related. In DR, ganglion cell degeneration occurs, which is one of the reasons for vision loss. To date, the pathogenesis of ganglion cell degeneration in DR remains incompletely understood. The presence of microglia activation and increased IDO and QUIN expression in T1D and T2D retinas may provide insight into a mechanism for neuronal loss in diabetes.

Activated retinal microglia previously have been found in donor diabetic retinal tissue.^53^ In this study, the density of Iba-1^+^ CD39^-^ microglia/macrophages in the retinas of T1D and T2D subjects was increased. Iba-1^+^/CD39^−^ microglia/macrophages exhibited an activated morphology when compared with those of nondiabetic donors (Fig. 1). In contrast, bright CD39^+^ resident microglia in the diabetic retinas were reduced in number (Fig. 1). CD39 has anti-inflammatory functions, and CD39 activity can attenuate microglia phagocytosis.^54^ CD39 overexpression has protective effects against STZ-induced diabetes in mice.^55^ Mice deficient in CD39 are highly susceptible to STZ-induced diabetes.^56^ Previously, we found increased Iba-1^+^/CD39^−^ microglia/macrophage and decreased CD39^+^ resident microglia densities in diabetic hypothalamus, which suggests activation of CNS inflammation.^57^ Increased expression of proinflammatory cytokines, such as interleukin-1β and tumor necrosis factor α (TNF-α) are observed in DR.^58^ The increased expression of the proinflammatory cytokines can, in turn, activate the KP, which has been shown to contribute to CNS pathology and neuronal loss.^6,12,50^

IDO activation triggers the KP. IDO expression is low or absent in the normal CNS,^59^ but can be induced in macrophages, microglia, neurons, astrocytes,^60,61^ and vascular endothelial cells.^62,63^ Increased enzyme expression can be provoked in neuro-inflammatory disorders, such as cerebral malaria.^12,59^ The increased IDO expression is believed to be mainly caused by the presence of T-lymphocytes and/or IFN-γ^59^ and other cytokines such as TNF.^64^ The predominant physiological inducer of IDO expression is IFN-γ. In T1D and T2D, sources of IFN-γ can be resident retinal cells as well as circulating cells that extravasate into the retina. Furthermore, IFN-γ is expressed locally in the CNS^65^ and activated microglia in the diabetic brain exhibit enhanced IFN-γ expression.^66^ Activated microglia produce TNF in experimental DR.^67^ Evidence in support of systemic production of relevant cytokines comes from the observation that there are high serum levels of IFN-γ and TNF in T1D subjects with DR.^47–49^

In this study, weak IDO expression was found in microglia-like cells and vascular endothelial cells in nondiabetic retinas (Figs. 2A, 2B). In contrast, strong IDO expression was found in T1D (Figs. 2C, 2D) and T2D (Figs. 2E, 2F) retinas. The quantitative data (Figs. 2G–I) indicated that IDO^+^ microglia-like cells in T1D and T2D retinas increased in density and brightness when compared with those of nondiabetic cells, but the brightness of IDO expression on vascular endothelial cells was similar in all three conditions. Our observation of IDO expression by microglia is consistent with the local production of IFN-γ and TNF.^68^ Furthermore, the loss of blood–retinal barrier function in DR would permit systemic IFN-γ and TNF to access the retinal parenchyma, promoting IDO activation.^69^

IDO is one of the prominent mediators of immune regulation.^51^ In the CNS, IDO expression has dual roles in immune responses.^8^ On the one hand, IDO can induce immune tolerance to down-regulate inflammation in several experimental autoimmune diseases in the CNS, including multiple sclerosis and experimental autoimmune encephalomyelitis.^70,71^ On the other hand, as the first enzyme in the KP, the up-regulation of IDO expression can increase downstream KP products, such as QUIN, which have the potential to promote immune-mediated neuronal damage.^8^ In the normal CNS, QUIN is only expressed on macrophage-like cells.^72^ However, in CNS inflammatory diseases, such as amyotrophic lateral sclerosis, expression of IDO and QUIN is increased on microglia and neurons.^61^

In this study, IDO and QUIN expressions were both increased on microglia-like cells in the diabetic human and rat retinas when compared with nondiabetic controls (Figs. 2, 3, 4), which suggested that the increased IDO^+^ expression by these cells might promote the expression of QUIN. Activated microglia are capable of producing the whole spectrum of KP metabolites, whereas astrocytes do not produce significant amounts of QUIN.^73^ Thus, our observation of IDO and QUIN in microglia in diabetic retinas suggests that these cells are the most important source of KP metabolites in this disease. Furthermore, the presence of IDO-negative, QUIN^+^ Müller cells (the major retinal microglia besides astrocytes)^74^ is consistent with the uptake of QUIN by these cells.

QUIN is an endogenous metabolite of the KP and is involved in several neuronal degenerative disorders, such as Alzheimer's disease and Huntington's disease.^75^ QUIN neural toxicity involves the following mechanisms: (1) continuous stimulation of *N*-methyl-d-aspartate receptors, with calcium entry into neurons^11^; (2) activation of second messenger-dependent protein kinases, which phosphorylate head domain sites on neurofilament subunits, potentially dysregulating intermediate filament assembly^76^; (3) impairment of the sarco/endoplasmic reticulum calcium-ATPase pump resulting in disturbed intracellular calcium signaling^77^; (4) increased glutamate release by neurons, inhibition of uptake by astrocytes, and inhibition of astrocyte glutamine synthesis, thereby increasing glutamate concentration in the microenvironment, leading to neurotoxicity^78^; (5) progressive energetic dysfunction, leading to neurodegeneration^79^; and (6) increased neuronal nitric oxide synthases, resulting in DNA damage, NAD(^+^) depletion, and neuronal death.^80^

Our study has demonstrated a marked reduction in the density of retinal neurons, as identified by NueN^+^ staining in the ganglion cell layer of the retinas of T1D and T2D compared to the nondiabetic controls (Fig. 7). Our findings are in agreement with clinical studies utilizing optical coherence tomography of the retina and demonstrating quantitative evidence of neurodegenerative changes in subjects with diabetes.^81^ Even in the absence of vascular changes, the retinal nerve fiber layer thickness around the optic disc is thinner in T2D subjects when compared with controls.^82^ Recently, Jeon et al.^83^ confirmed these findings and demonstrated that average retinal nerve fiber layer thickness in T2D individuals was significantly thinner than that of nondiabetic, nonglaucomatous controls. These changes were present in individuals with no DR or mild nonproliferative retinopathy. It was interesting to note in this study that QUIN^+^ expression in human T1D retinas was significantly higher than that in T2D retinas (Fig. 4), whereas the density of NeuN^+^ neurons in human T1D retinas was significantly lower than that in T2D retinas (Fig. 7), which suggests that the loss of NeuN^+^ neurons may be related to the increased QUIN^+^ expression in human diabetic retinas.

**Figure 8:**
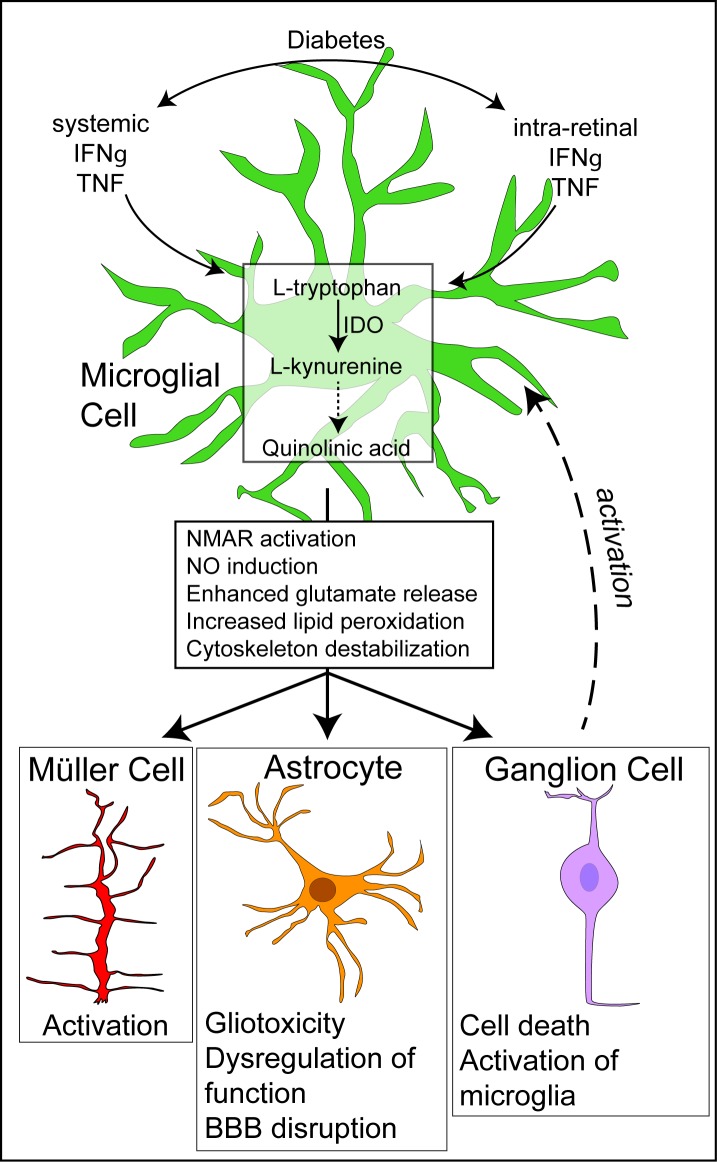
A schematic showing the role of the kynurenine pathway in ganglion cell degeneration and inflammation in T1D and T2D. Our study showed increased IDO and QUIN expression in the microglia and Müller cells of diabetic human and rodent retina. Diabetes leads to increased IFN-γ and TNF both systemically and in the retina. These cytokines induce IDO expression in microglia, leading to toxic production of QUIN. The QUIN causes dysfunction of astrocytes and the retinal blood barrier, as well as ganglion cell death, which in turn leads to microglial activation. QUIN also is taken up by Müller cells, possibly leading to their activation.

In summary (Fig. 8), increased densities of Iba-1^+^ microglia and Iba-1^+^/CD39^-^ bone marrow–derived macrophages occur in human T1D and T2D and diabetic rodent retinas, demonstrating the existence of local inflammation. CD39 has anti-inflammatory and protective functions. Reduced CD39 expression on Iba-1^+^ microglia may lead to increased production of proinflammatory cytokines, in particular IFN-γ, which induce IDO expression and activity. Enhanced microglial IDO activity increases the generation of QUIN via the KP. QUIN can increase the extracellular glutamate concentration and induce neuronal nitric oxide synthesis, resulting in DNA damage and NAD(^+^) depletion. QUIN also may adversely affect blood–brain barrier function and lead to astrocyte dysfunction and damage, thereby contributing to neuronal injury. All of these mechanisms could potentially contribute to neuronal loss in diabetic retinas (Fig. 7).
